# *SIMBAD*: a sequence-independent molecular-replacement pipeline

**DOI:** 10.1107/S2059798318005752

**Published:** 2018-06-08

**Authors:** Adam J. Simpkin, Felix Simkovic, Jens M. H. Thomas, Martin Savko, Andrey Lebedev, Ville Uski, Charles Ballard, Marcin Wojdyr, Rui Wu, Ruslan Sanishvili, Yibin Xu, María-Natalia Lisa, Alejandro Buschiazzo, William Shepard, Daniel J. Rigden, Ronan M. Keegan

**Affiliations:** a Institute of Integrative Biology, University of Liverpool, Liverpool L69 7ZB, England; b Synchrotron SOLEIL, L’Orme des Merisiers, Saint Aubin, BP 48, 91192 Gif-sur-Yvette, France; c STFC, Rutherford Appleton Laboratory, Harwell Oxford, Didcot OX11 0FA, England; d CCP4, Research Complex at Harwell, Rutherford Appleton Laboratory, Harwell Oxford, Didcot OX11 0FA, England; e Global Phasing Ltd, Cambridge CB3 0AX, England; f Feil Family Brain and Mind Institute, Weill Cornell Medicine, New York, NY 10021, USA; g GM/CA@APS, The X-Ray Science Division, The Advanced Photon Source, Argonne National Laboratory, Lemont, IL 60439, USA; h Division of Structural Biology, Walter and Eliza Hall Institute of Medical Research, Parkville, VIC 3052, Australia; i Department of Medical Biology, University of Melbourne, Royal Parade, Parkville, VIC 3050, Australia; j Laboratory of Molecular and Structural Microbiology, Institut Pasteur de Montevideo, Mataojo 2020, 11400 Montevideo, Uruguay

**Keywords:** molecular replacement pipeline, *SIMBAD*, contaminant, lattice search, structure solution

## Abstract

The conventional approach to finding structurally similar search models for use in molecular replacement (MR) is to use the sequence of the target to search against those of a set of known structures. Sequence similarity often correlates with structure similarity. Given sufficient similarity, a known structure correctly positioned in the target cell by the MR process can provide an approximation to the unknown phases of the target. An alternative approach to identifying homologous structures suitable for MR is to exploit the measured data directly, comparing the lattice parameters or the experimentally derived structure-factor amplitudes with those of known structures. Here, *SIMBAD*, a new sequence-independent MR pipeline which implements these approaches, is presented. *SIMBAD* can identify cases of contaminant crystallization and other mishaps such as mistaken identity (swapped crystallization trays), as well as solving unsequenced targets and providing a brute-force approach where sequence-dependent search-model identification may be nontrivial, for example because of conformational diversity among identifiable homologues. The program implements a three-step pipeline to efficiently identify a suitable search model in a database of known structures. The first step performs a lattice-parameter search against the entire Protein Data Bank (PDB), rapidly determining whether or not a homologue exists in the same crystal form. The second step is designed to screen the target data for the presence of a crystallized contaminant, a not uncommon occurrence in macromolecular crystallography. Solving structures with MR in such cases can remain problematic for many years, since the search models, which are assumed to be similar to the structure of interest, are not necessarily related to the structures that have actually crystallized. To cater for this eventuality, *SIMBAD* rapidly screens the data against a database of known contaminant structures. Where the first two steps fail to yield a solution, a final step in *SIMBAD* can be invoked to perform a brute-force search of a nonredundant PDB database provided by the *MoRDa* MR software. Through early-access usage of *SIMBAD*, this approach has solved novel cases that have otherwise proved difficult to solve.

## Abstract 2

*SIMBAD* is a sequence-independent molecular-replacement pipeline for solving difficult molecular-replacement cases where contaminants have been crystallized. It can also be used to find structurally related search models where no obvious homologue can be found through sequence-based searching.

## 1. Introduction   

In X-ray crystallography, the problem of solving the three-dimensional structure of a protein remains a difficult task. Even with crystals diffracting to high resolution, many projects flounder owing to the challenges involved in overcoming the phase problem. For macromolecules with more than a few hundred atoms, solving the phase problem directly is currently not viable, so an alternative approach must be used. Molecular replacement (MR) is the most popular route to solve the problem as it is quick, inexpensive and can be highly automated (Evans & McCoy, 2008 ▸; Long *et al.*, 2008 ▸). MR exploits the fact that proteins with similar amino-acid sequences typically form similar three-dimensional structures. Where a known structure has a similar sequence to a target, the phase information from the known structure can, assuming that there is corresponding structural similarity, often be used as a starting point for the phases of the unknown structure. The procedure requires that the known structure is reorientated and positioned correctly in the unit cell of the target. Programs incorporating sophisticated scoring systems such as *Phaser* (McCoy *et al.*, 2007 ▸) and *MOLREP* (Vagin & Teplyakov, 2010 ▸) have been developed to perform this task. However, the selection of an appropriate search model remains a limiting factor in MR. Sequence similarity does not always ensure structural similarity, particularly where the similarity is lower than 30% (Krissinel & Henrick, 2004 ▸; Krissinel, 2007 ▸). Some recent studies have sought alternative ways of finding structurally similar search models. Approximating target structures through *ab initio* modelling and using these as search models has been shown to work by Qian *et al.* (2007 ▸) and Rigden *et al.* (2008 ▸) and can be exploited using the *AMPLE* application (Bibby *et al.*, 2012 ▸). Other approaches make use of idealized fragments or regularly occurring fragments and motifs from known structures as search models in MR. *ARCIMBOLDO* (Rodríguez *et al.*, 2009 ▸) and *Fragon* (Jenkins, 2018 ▸) are two developments exploiting this approach. All of these applications mainly rely on small but highly accurate fragments being placed correctly in the unit cell of the target. In the most extreme cases, where data are available to 1 Å resolution or better, it has been shown that it is possible to use single atoms as a successful search model (McCoy *et al.*, 2017 ▸).

For the more traditional sequence-based approach, much effort has been put into developing software pipelines that will attempt to find a solution from a large set of carefully crafted search models from potentially suitable homologues. Examples of these include *MoRDa* (Vagin & Lebedev, 2015 ▸), *MrBUMP* (Keegan *et al.*, 2018 ▸), *BALBES* (Long *et al.*, 2008 ▸) and *MRage* (McCoy *et al.*, 2007 ▸). The search models selected by these applications or manually by a user can give poor results for a number of reasons. These include insensitivity of the template search (*i.e.* the homologue is too divergent from the actual structure), misleading sequence information (*i.e.* a contaminant has been crystallized in place of the desired protein) or the sequence similarity providing an imperfect proxy for structural similarity (*i.e.* where relatives with high sequence similarity have been crystallized in different conformational states). In such cases, *ARCIMBOLDO* and *Fragon* may retrieve the solution through the correct placement of idealized fragments such as helices, but are limited by the resolution requirements of *SHELXE* (∼2.4 Å; Thorn & Sheldrick, 2013 ▸) and *ACORN* (∼1.7 Å; Foadi *et al.*, 2000 ▸; Yao *et al.*, 2005 ▸), respectively, when improving upon the phases given by the initial placement of the fragment by *Phaser*. Some developments have sought to overcome these problems by attempting to unearth suitable search models through a brute-force search of the PDB (Stokes-Rees & Sliz, 2010 ▸; Hatti *et al.*, 2016 ▸). *ContaMiner* (Hungler *et al.*, 2016 ▸) is another approach specifically aimed at finding contaminants by testing a library of known contaminants in MR.

Here, we present a new pipeline, *SIMBAD* (*Sequence-Independent Molecular replacement Based on Available Databases*), which can be used for both contaminant and brute-force approaches. Its ability to detect contaminant crystal structures is relevant to cases such as Keegan *et al.* (2016 ▸), where the structure remained unsolved for 14 years. It ensures acceptably low run times by testing only the non­redundant PDB entries as defined in the *MoRDa* database and shortcutting the process by testing first for crystals with a familiar unit cell or containing known contaminants. *MoRDa* is a conventional MR pipeline built upon the *MOLREP* program. Its database contains chains from a redundancy-removed version of the PDB database and definitions of how to construct domains, oligomers, complexes and ensembles from the individual chains. In its current implementation, *SIMBAD* uses only the domain definitions to create search models. In total, *SIMBAD* contains three steps: a lattice-parameter search, a contaminant search and the non­redundant PDB *MoRDa* database search (henceforth referred to as the *MoRDa* DB search). Each can be run as a separate module, with the complete run involving all three steps being referred to as the combined search.

In the absence of relevant sequence-identity information to help isolate and score suitable search models, *SIMBAD* makes use of the rotation-function step in MR to rank search models ahead of performing a full MR search. The rotation function is a three-dimensional search used to determine the proper orientation of a search model. It was first discussed in the context of the self-Patterson by Hoppe (1957 ▸) and Huber (1965 ▸). However, the rotation function that we know today was first proposed by Rossmann & Blow (1962 ▸). This initial rotation function exploited noncrystallographic symmetry to recover the phases required for structure determination. Rossmann and Blow also recognized that this concept could be applied to the problem of positioning a known molecule in an unknown crystal lattice by applying an additional translation procedure. The rotation search was first applied in this context by Crowther & Blow (1967 ▸). The original rotation function was a slow calculation. Crowther expanded the Patterson functions in terms of spherical harmonics and spherical Bessel functions to create the fast rotation function (Crowther, 1972 ▸). Navaza further refined the fast rotation function to use a numerical integration rule in place of expansions in the radial function (Navaza, 1987 ▸). It was this version of the rotation function that was incorporated into *AMoRe* (Navaza, 1993 ▸).

More recently, Read began exploring maximum-likelihood methods as an alternative way to approach the rotation function (Read, 2001 ▸). An initial implementation added to *Beast* (Read, 1999 ▸, 2001 ▸) demonstrated an increase in sensitivity compared with Patterson-based rotation functions when applied to difficult cases. This initial maximum-likelihood method was a slow calculation. Storoni and coworkers introduced a likelihood-enhanced fast rotation function for implementation in *Phaser* (Storoni *et al.*, 2004 ▸). The likelihood-enhanced fast rotation function utilizes series approximations to the full likelihood target that can be calculated quickly by the fast Fourier transform. This approximation of the full likelihood target improves the speed by several orders of magnitude. More recently, Caliandro and coworkers developed a probabilistic approach to the rotation problem in *RENO*09 (Caliandro *et al.*, 2009 ▸). Similarly to the maximum-likelihood methods already discussed, the probabilistic approach constructs probability distributions for a rotated model in a given environment, although the final formulas derived differ from those obtained *via* maximum-likelihood principles.

*SIMBAD* performs the rotation search ∼90 000 times when screening the full *MoRDa* DB and, as such, speed and efficiency are very important. In light of this, the *AMoRe* rotation function was selected, as the modular nature of the program allowed us to pre-calculate a spherical harmonic coefficient database from the 90 000 models, a prerequisite for the rotation search. Ultimately this approach was not adopted, but it was the initial motivation behind the selection of *AMoRe*. However, the speed of the *AMoRe* rotation function (of the order of seconds) made the processing of such large numbers of search models tractable on a modest cluster.

In all cases the best matches are tested by MR and refinement to ascertain whether or not they give a solution. *SIMBAD* can make use of multi-core clusters to speed up its processing of search models, enabling its combined three-step functionality to be run, for example, in the space of a few hours on a 100-core machine (2.8 GHz, AMD Opteron 4184). The software is distributed with the *CCP*4 suite (Winn *et al.*, 2011 ▸) and will be made available through the *CCP*4 online/cloud developments in the future. It can also be run as part of the data-processing pipelines at synchrotron beamlines to test for the presence of contaminants early in the structure-solution process.

## 2. Methodology   

### 2.1. Strategy   

**Figure 1:**
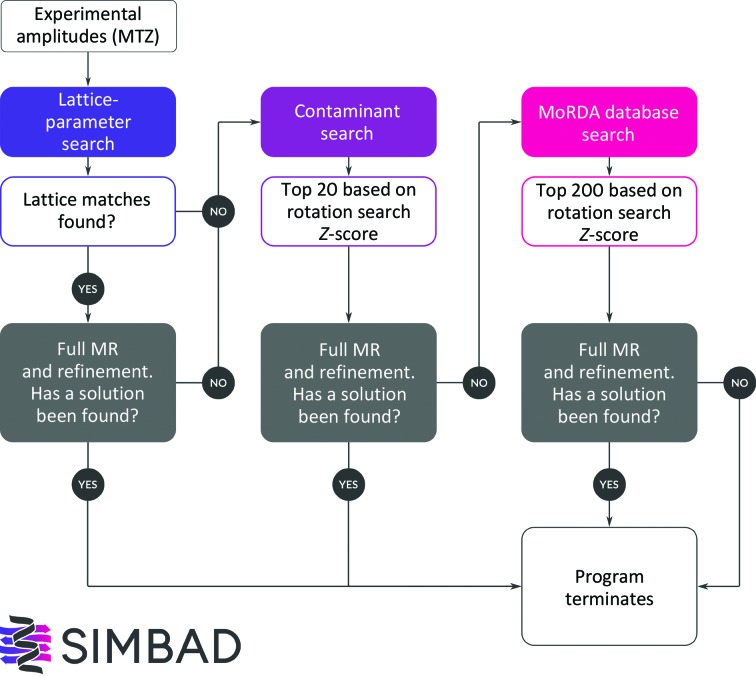
Flowchart detailing the decision processes in the *SIMBAD* pipeline. The Full MR step in each case refers to performing a complete MR procedure (rotation and translation search) using the best-ranked models from the initial search (lattice-parameter, contaminant or *MoRDa* DB).

A flowchart of the *SIMBAD* pipeline is presented in Fig. 1 ▸. Within the three-step procedure of *SIMBAD*, two different methods are used to identify unknown crystals independently of sequence. The first method searches for structures in the PDB with similar lattice parameters to the unknown structure. Similar lattice parameters often indicate that a different, previously characterized protein has been crystallized by mistake (Niedzialkowska *et al.*, 2016 ▸). The second method exploits the *AMoRe* (Navaza, 1994 ▸) rotation search to screen a database of candidate search models. This is split into two steps. The first step consists of screening a small database of structures that have been identified to commonly contaminate crystals. The second step consists of screening the full *MoRDa* DB. The *MoRDa* DB run is by far the most computationally expensive step and therefore the lattice-parameter/contaminant searches are run first.

### 2.2. Lattice-parameter search   

The *SIMBAD* lattice-parameter search employs a similar strategy to that used by the *Nearest-cell* server (Ramraj *et al.*, 2012 ▸) and the *SAUC* server (McGill *et al.*, 2014 ▸). A database was created from the PDB containing the Niggli reduced cell, a reduced *P*1 cell (Andrews & Bernstein, 2014 ▸), for each structure using the explore_metric_symmetry routine in *cctbx* (*Computational Crystallography Toolbox*; https://github.com/cctbx/cctbx_project). The Niggli reduced cell for the unknown data set is generated in the same way and compared with the Niggli reduced cells in the database.

The comparison takes place in two steps. Firstly, the Niggli reduced-cell database is searched for cells where each lattice parameter is within ±5% of the respective lattice parameter in the experimental data. Secondly, a penalty score is generated for each Niggli reduced cell using
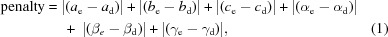
where *a*, *b* and *c* represent the lengths of the cell edges and α, β and γ represent the angles between them. A subscript e signifies experimentally derived lattice parameters and a subscript d is used for Niggli reduced-cell database-derived lattice parameters.

**Figure 2:**
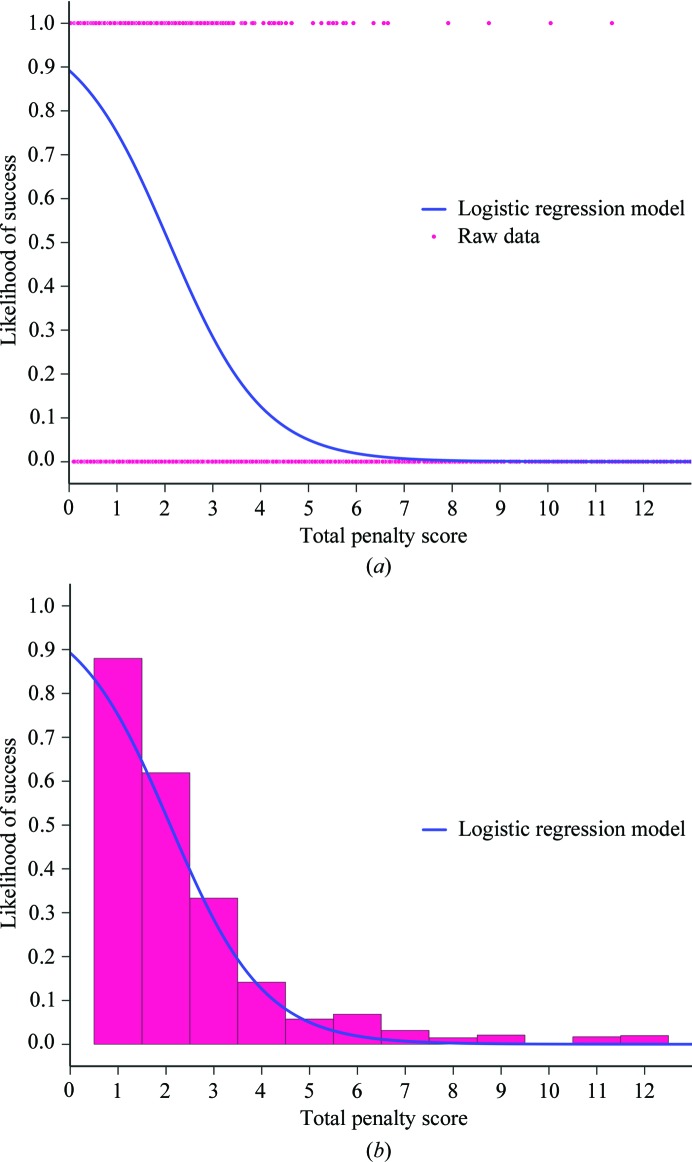
Logistic regression results showing the likelihood that a penalty score would result in successful MR. The purple line describing the distribution was fitted using a sigmoid model. The coefficient and intercept were determined by the ‘LogisticRegression’ module in *sklearn* (http://www.scikit-learn.org). (*a*) The scatter points represent the 2009 raw data points, where the *x* value corresponds to the total penalty score and the *y* value is set to 1 or 0 to indicate success or failure in MR. (*b*) The histogram represents the proportion of success/failure for bin sizes of 1. The figure has been truncated to show the results up to a penalty score of 13; however, the sigmoid model was calculated from data sets with penalty scores of up to 26.

To test the intuition that a lower penalty score would be more likely to lead to a solution, a test set of 125 data sets were randomly selected from the PDB (Supplementary Table S1). By performing the lattice-parameter search on each of these data sets, a total of 2009 unique candidates with varying penalty scores were obtained. For each candidate, MR and refinement were carried out against the relevant data set using *MOLREP* and *REFMAC*5 (Murshudov *et al.*, 2011 ▸). A search model/penalty score was considered to have given a solution if the *R*_free_ fell below 0.45. These data were used to train a logistic regression classifier (Fig. 2 ▸). The training was used to fit a sigmoid function to the data, giving the equation
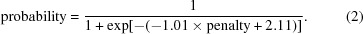



The accuracy with which the model predicted whether a candidate search model would lead to success in MR was evaluated at 87% on the test set, matching the 87% on the training set (Supplementary Table S2). This model has been implemented into *SIMBAD* to give users an indication of whether a candidate is likely to return a solution.

Our model suggests that below a penalty score of 2.1 the probability of finding a solution exceeds 50%. In our data set, not a single example was found where a penalty score above 12 returned a solution. Therefore, the lattice-parameter search was set to return up to 50 models with penalty scores below 12 by default.

### 2.3. Rotational search   

*SIMBAD* uses the *AMoRe* fast rotation function to screen databases for suitable MR candidates. By skipping models estimated to be unable to fit into the unit cell (by requiring a solvent content above 30%) and by exploiting coarse-grained parallelization across a multi-CPU cluster, the time required for the rotation function is minimized. *SIMBAD* uses the correlation co­efficient between the observed amplitudes for the crystal and the calculated amplitudes for the model (CC_F) to score the results from *AMoRe*. A large peak in the CC_F score for the top-ranked solution is indicative of a correctly orientated structure. Therefore, in order to compare the solutions for each template structure used, *AMoRe* was modified to return a *Z*-score of the CC_F scores. The *AMoRe* *ROTNDO* sub­routine was modified to output *Z*-scores derived from CC_F and the correlation map. The CC_F-based *Z*-score estimates the mean and variance for the template using 200 random orientations.

#### 2.3.1. Contaminant search   

A set of 349 structures representing the different homologues and space groups of 60 proteins known to commonly occur as contaminants has been compiled. This set consists of contaminants identified in the course of developing *SIMBAD* and common contaminants listed by other sources (Niedzialkowska *et al.*, 2016 ▸; Hungler *et al.*, 2016 ▸). In addition, corresponding domains from the *MoRDa* DB which may form subcomponents of the contaminants augment the original database. The complete list is processed in the *AMoRe* rotation search and the models are ranked by *Z*-score. The top 20 are passed on to *MOLREP* and *REFMAC*5 for full MR and refinement.

#### 2.3.2. *MoRDa* DB search   

The *MoRDa* DB step of *SIMBAD* screens the *MoRDa* DB for potential MR templates. *MoRDa* includes its own edited version of the PDB which contains a nonredundant domain database of ∼90 000 domains (at the time of this study). The *SIMBAD* pipeline processes the entire set using the fast *AMoRe* rotation search. The models are used as they are defined in the *MoRDa* database with no additional modifications. Each is then ranked by *Z*-score and the top 200 solutions are passed on to *MOLREP* followed by *REFMAC*5 to perform full MR and refinement. Based on preliminary testing, this figure of 200 was able to catch some nontrivial cases. Subsequent work showed it to strike a good balance between speed and sensitivity, although it has not been extensively tested.

### 2.4. Full MR and refinement   

The final step in each of the lattice-parameter, contaminant and *MoRDa* DB searches is to process the best scoring matches using first *MOLREP* to perform a full MR search and then *REFMAC*5 to refine the resulting positioned model. By default, *REFMAC*5 performs 30 cycles of restrained refinement for the lattice-parameter and contaminant searches and 100 cycles of restrained refinement for the *MoRDa* DB search. Defaults are used for all other parameters in both programs. The results are presented to the user *via* *jsrview* (Krissinel *et al.*, 2018 ▸), a report-generating tool distributed with *CCP*4. Tables of scores and plots of *R*/*R*_free_ statistics sorted by the final *R*_free_ value after refinement are presented to the user. An *R*_free_ of 0.45 is suggested as indicative of a solution, but the user may also examine maps and positioned models. When *SIMBAD* is run locally this can be performed using *Coot* (Emsley *et al.*, 2010 ▸). When executed online, the molecular-graphics tool *UglyMol* (https://github.com/uglymol) is used instead. The *Z*-scores from the *AMoRe* rotation search for the contaminant and *MoRDa* DB stages are also made available. Supplementary Fig. S1 shows the report page for a run of *SIMBAD*.

## 3. Results   

### 3.1. Testing the *SIMBAD* pipeline   

The first two steps of *SIMBAD*, the lattice-parameter and contaminant searches, are quick but thorough approaches to find search models that are suitable for MR in cases where a contaminant is present or when a related structure with very similar cell dimensions is available. Invoking these two options on their own is well suited for use as a post-data-collection rapid screening of data sets to ensure that a contaminant is not present. The follow-on step of screening the entire *MoRDa* DB for possible search models can, in addition to finding cases of new contaminants or misidentification, offer the possibility of finding a non-obvious search model for a novel target structure.

To realistically evaluate the capabilities of *SIMBAD*, we conducted two sets of tests. Firstly, we tested its ability to find contaminants through its lattice-parameter and contaminant searches. A second set of tests was designed to investigate how readily it can find a suitable search model from the *MoRDa* DB for use in determining the solution of a novel structure.

#### 3.1.1. Testing for contaminant structure solution   

The two main routes to identify the presence of a known contaminant are through the lattice-parameter search or, where this fails, through explicitly testing each entry in our contaminant list *via* the *AMoRe* rotational search. The former has the advantage of speed but relies on the contaminant crystallizing in an almost identical unit cell. The latter is more thorough but takes longer. Test results for the lattice-parameter search on simulated novel structures are given in the following section. Here, we present the results of testing the contaminant search.

In order to simulate a scenario in which a contaminant had been crystallized in a new space group/unit cell, ten structures were selected that represented a unique space group among a subset of homologues in our contaminant list. These structures were removed from our database to determine whether the contaminant search would be successful in identifying homologues in other space groups as suitable candidates for MR search models. The ten cases represented a broad range of space groups, resolutions and structure types.

**Figure 3:**
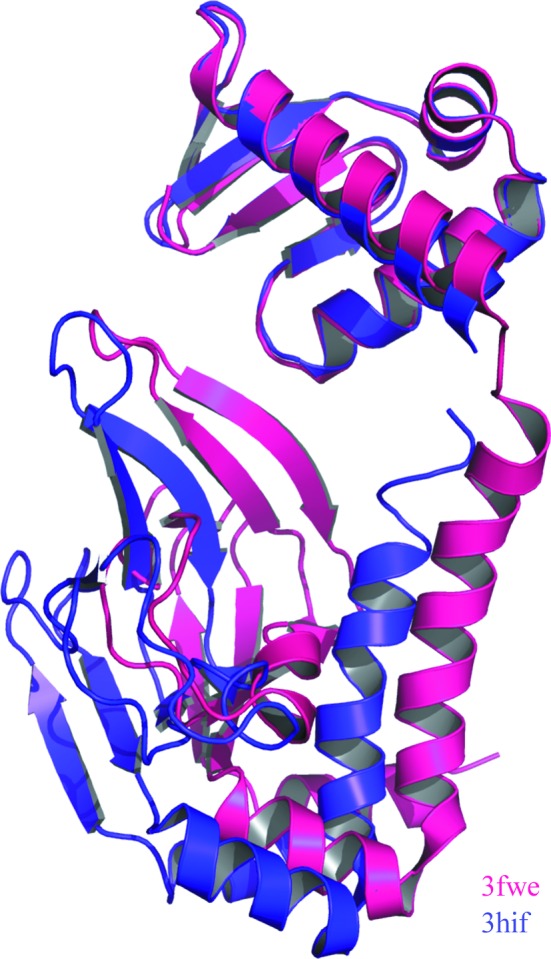
Structural alignment of the C-terminal DNA-binding domains of the apo D138L CAP mutant (PDB entry 3fwe) chain *B* (pink) and apo wild-type CAP (PDB entry 3hif) chain *B* (purple), highlighting the conformational change.

*SIMBAD* was successful in nine out of the ten test cases (Supplementary Table S3). Analysis of the failed case (PDB entry 3fwe, an apo D138L CAP mutant) showed that the homologues for this structure had significantly larger conformational differences than for the nine successful cases. The conformational differences were measured using the pairwise structural alignment feature in *GESAMT* (Krissinel, 2012 ▸). The best search models were compared with the targets in terms of a C^α^ r.m.s.d. and a *Q*-score. For the nine cases that succeeded the average C^α^ r.m.s.d. and *Q*-score were 0.51 and 0.89, respectively, and for the one case that failed the closest match in the contaminant database (PDB entry 3hif) only gave a C^α^ r.m.s.d. and *Q*-score of 1.56 and 0.75, respectively. This model was ranked 172nd, with a *Z*-score of 3.2. It has been shown that apo wild-type CAP (PDB entry 3hif) undergoes large conformational changes in order to bind DNA (Sharma *et al.*, 2009 ▸). Such conformational changes would explain the intramolecular differences seen between the apo D138L CAP mutant (PDB entry 3fwe) and apo wild-type CAP (PDB entry 3hif) (Fig. 3 ▸).

In conclusion, *SIMBAD* is able to identify contaminants that are crystallized in a similar unit cell to existing structures using the lattice-parameter search but is also able to identify contaminants crystallized in novel ways when a sufficiently similar (C^α^ r.m.s.d. < 1 Å) structure is contained within our contaminant database.

#### 3.1.2. Testing for novel structure solution   

To simulate cases where the sequence is potentially unknown for a given target, we tested the *SIMBAD* combined search (lattice-parameter, contaminant and *MoRDa* DB searches) against a set of 25 recently released structures in the PDB. These cases were all released in February or March 2017. The *SIMBAD* lattice database and the version of the *MoRDa* DB used at the time of testing did not contain any entries with information derived from this set of PDB structures or any subsequently released PDB entries. Other than this criterion, no particular constraints were placed on the PDB entries chosen. The set contained a wide range of resolution limits, numbers of copies in the asymmetric unit, space groups, monomer sizes and secondary-structure types (Supplementary Table S4). It also included cases that were originally solved by MR, SAD, MAD and SIRAS methods. The results of the testing are presented in Supplementary Table S4. *SIMBAD* was successful in 13 of the 25 test cases, a success rate of 52%. Solutions were verified by a map correlation coefficient (map CC) with an electron-density map generated for the deposited data using *phenix.get_cc_mtz_mtz* (Adams *et al.*, 2010 ▸). Correct solutions had a mean map CC of 0.88. Six cases were solved by the lattice-parameter search, with the remaining seven being solved by the *MoRDa* DB search.

One of the goals of our tests was to examine the degree of similarity between the model and target that was required in order to produce a solution. To this end, we examined the similarity between the top-scoring successful search model and its respective target in three different ways for each of the 25 cases. Firstly, we looked at the sequence identity. The mean sequence identity of a successful search model to the target was 98% in the lattice-parameter search and 83% in the *MoRDa* DB search. The lowest sequence identity between a successful search model and the target was 44% [PDB entry 5grh using search model 3blvA_1 (*MoRDa* DB format: PDB code 3blv, chain *A*, domain 1)]. We then examined the coverage of the target structure by the search model. The search model with the smallest relative size to the target was 3jwnH_2, making up approximately 14% of the overall content of the asymmetric unit of PDB entry 5jqi (eight chains, 1157 residues in total). This model ranked top in the *MoRDa* DB search and had 100% sequence identity to the part of the target matched. On average, a successful search model made up 44% of the content of the asymmetric unit of the target. Finally, by utilizing the pairwise structural alignment feature in *GESAMT*, we compared the search models with the targets in terms of a C^α^ r.m.s.d. and a *Q*-score (a measure of structural similarity, where 1 is identical and 0 is structurally unrelated). Results for successful solutions showed an average C^α^ r.m.s.d.s and *Q*-scores of 0.63 and 0.93, respectively, for the lattice-parameter search and 0.61 and 0.46, respectively, for the *MoRDa* DB search. The highest C^α^ r.m.s.d. between the model and the target for a success was 0.88 Å (PDB entry 5mg1) in the lattice-parameter search and 1.08 Å (PDB entry 5grh) in the *MoRDa* DB search. The *MoRDa* DB search ranked this model 35th, with a *Z*-score of 5.6.

In conclusion, within our test set *SIMBAD* was capable of producing MR solutions using search models that are significantly different from the target in terms of sequence identity (≥44%), model coverage (≥14%) and C^α^ r.m.s.d. (≤1.07 Å). This demonstrates the usefulness of *SIMBAD* for more than just known contaminant detection, showing it to be capable of finding solutions to novel structures where some search model is available with characteristics within the thresholds outlined above and possibly beyond. Notably, the resolution of the experimental data did not influence the ability to find a solution. Successful cases had resolutions in the range 1.5–3.3 Å.

As a follow-on to the above examination, we looked at the ability of *SIMBAD* to pick out a possible search model from the *MoRDa* DB given the availability of a structure within a C^α^ r.m.s.d. threshold of 1.07 Å. A *GESAMT* archive search of the *MoRDa* DB revealed that *SIMBAD* failed in only four of the 17 cases where there is some structure in the *MoRDa* DB that is within a 1.07 Å C^α^ r.m.s.d. of the target structure (assuming a minimum alignment to 30% of the target). Of the four cases that did not produce a solution, three (PDB entries 5lnl, 5jfm and 5ayl) were multi-chain or multi-domain targets of at least seven domains. The *MoRDa* models most closely matching these targets provided too small a signal for them to be found in the *AMoRe* rotation-search step. The remaining case (PDB entry 5hsm) had a single chain of 131 residues and the best *MoRDa* model (3fm5A_1, C^α^ r.m.s.d. = 0.97 Å) failed to produce a solution in *SIMBAD*. This model provided a weak signal in the rotation search (*Z* = 4) and was relegated to a low overall ranking by many similar, but higher scoring search models containing longer α-helices. In the cases where a successful solution was found using the *MoRDa* DB search, the resulting best search model was ranked top on three occasions. The lowest *AMoRe* ranking for a successful search model was 170. With this step trialling more than 90 000 search models, it demonstrates the sensitivity of the *Z*-scoring added to *AMoRe* but also the value of taking at least the top 200 ranked hits to the full MR and refinement stage. The *Z*-score values for successful solutions ranged from 5.5 (PDB entry 5uqf) to 14.0 (PDB entry 5uca) with a mean of 8.9.

Finally, we looked at the run times for the various test cases. The average run time for success during the lattice-parameter step was 0.7 h on a maximum of 20 cores (2.8 GHz, AMD Opteron 4184). Completion of the combined search required an average of 11.6 h on 40 cores, regardless of success or failure.

### 3.2. User cases   

In this section, we present three cases in which *SIMBAD* has been used to determine a difficult-to-solve case owing to the unwitting crystallization of a contaminant. Although the targets were ultimately of low importance for the structural insights that they provided, their solution prevented further misdirected effort on the part of the researchers involved. All cases involve the crystallization of a known contaminant. Examples involving the use of *SIMBAD* for novel structure solution are available elsewhere, such as PDB entries 6byq, 6c87 and 5wol. Cases illustrating the use of *SIMBAD* for targets that had not been previously sequenced are not shown owing to the publications being in progress at the time of writing. Solutions for mislabelled crystals are also not shown. These cases were of little interest to the researcher once the mistake had been realized, and no further effort was devoted to structure completion.

#### 3.2.1. *Escherichia coli* DPS protein contaminant   

Crystals of the contaminant protein DPS (DNA-protecting protein during starvation) grew in previously established conditions for caspase 1: the vapour-diffusion method with a well solution consisting of 0.1 *M* sodium chloride, 0.1 *M* bis-tris pH 6.5, 1.5 *M* ammonium sulfate and hanging drops composed of a 1:1 mixture of the well solution and 8 mg ml^−1^ protein in a buffer consisting of 50 m*M* sodium acetate pH 5.9, 100 m*M* NaCl, 5% glycerol (R. Wu, unpublished results). Crystals did not grow in the expected time range, but appeared after several months at ambient temperature. They were cryoprotected by the addition of 20% glycerol to the well solution and cryocooled in liquid nitrogen. The crystals belonged to space group *C*222_1_, with lattice parameters *a* = 117.62, *b* = 133.97, *c* = 139.11 Å, α = β = γ = 90° and a presumed six molecules of caspase 1 in the asymmetric unit. Diffraction data were measured at 100 K using a PILATUS3 6M detector (Dectris) on the 23ID-D beamline of GMCA@APS at the Advanced Photon Source, Argonne National Laboratory, USA. The data were indexed, integrated and reduced with *XDS* (Kabsch, 2010 ▸).

**Figure 4:**
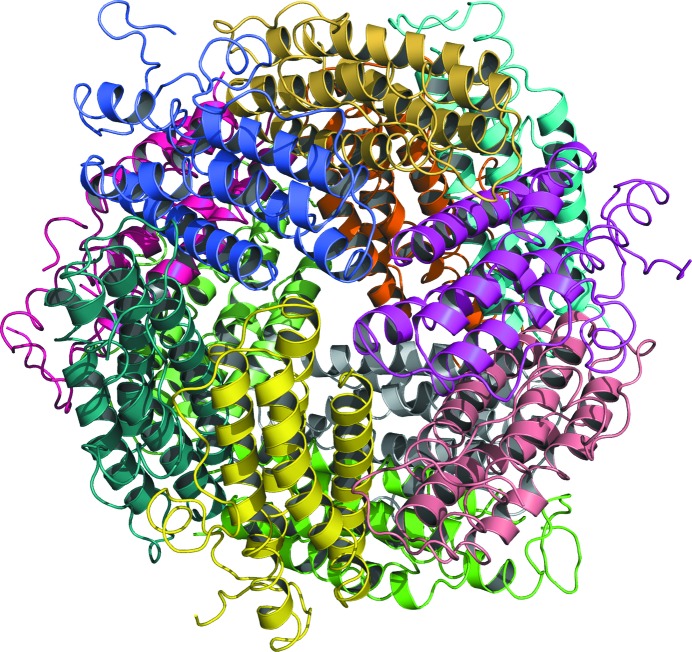
Cartoon representation of the *E. coli* DPS dodecamer, with protomers identified by colour.

The *SIMBAD* *MoRDa* DB search led to success with the structure of a 167-residue protein identified as a DNA-protecting protein during starvation from *E. coli* (PDB entry 1f30), which is characterized as a ferritin-like protein in the SCOP database (Murzin *et al.*, 1995 ▸). After refinement, it became clear that this was the protein that had crystallized instead of caspase 1. The structure of DPS was refined with *REFMAC*5 in the *CCP*4 suite to 1.5 Å resolution, resulting in *R* and *R*_free_ values of 17.64% and 20.77%, respectively. Manual model inspection and modifications were performed with *Coot*. In the crystal, 12 molecules of the protein form a hollow sphere closely reminiscent of that formed in crystals of ferritin (Fig. 4 ▸). The coordinates and structure factors have been deposited in the Protein Data Bank with accession code 6b0d and the raw data have been deposited in SBGrid (Morin *et al.*, 2013 ▸).

Caspase 1 had previously been successfully purified and crystallized, and its structure had been solved using MR (R. Wu, unpublished results). While there were telltale signs of possible contamination of the new protein preparation, they were not clear enough or had plausible alternative explan­ations. For example, the crystals from the current protein sample looked different from those used in the structure solution of caspase 1 and had very different unit-cell parameters. However, this was attributed to the fact that caspase 1 was cross-linked in the current sample. It was considered possible that the cross-linking might have interfered with the proper folding, since caspase 1 folds from two peptides in a two-step process. Therefore, it was thought that perhaps the final product was structurally significantly different from the molecule whose structure was solved previously. Initial difficulties in MR were attributed to the same possibility.

#### 3.2.2. *Serratia proteamaculans* cyanase protein contaminant   

Crystals of the contaminant protein (cyanase) grew in conditions expected to crystallize a cytokine complex: the vapour-diffusion method with a well solution consisting of 0.1 *M* magnesium acetate, 10% PEG 10K, 0.1 *M* MES pH 6.5 and hanging drops composed of a 1:1 mixture of the well solution and the protein complex. Crystals appeared after six months at ambient temperature. The crystals were cryoprotected with 20% ethylene glycol. The crystals belonged to space group *C*121, with lattice parameters *a* = 136.56, *b* = 94.13, *c* = 89.11 Å, α = 90, β = 125.49, γ = 90° and five molecules in the asymmetric unit. Diffraction data were collected using an ADSC Q315 detector on the MX2 beamline at the Australian Synchrotron. The data were indexed, integrated and reduced with *XDS*.

The *SIMBAD* *MoRDa* DB search led to successful structure solution with the 156-residue cyanase from *S. protea­maculans* (PDB entry 4y42). After refinement it became clear that this was the protein that had crystallized instead of the cytokine complex.

**Figure 5:**
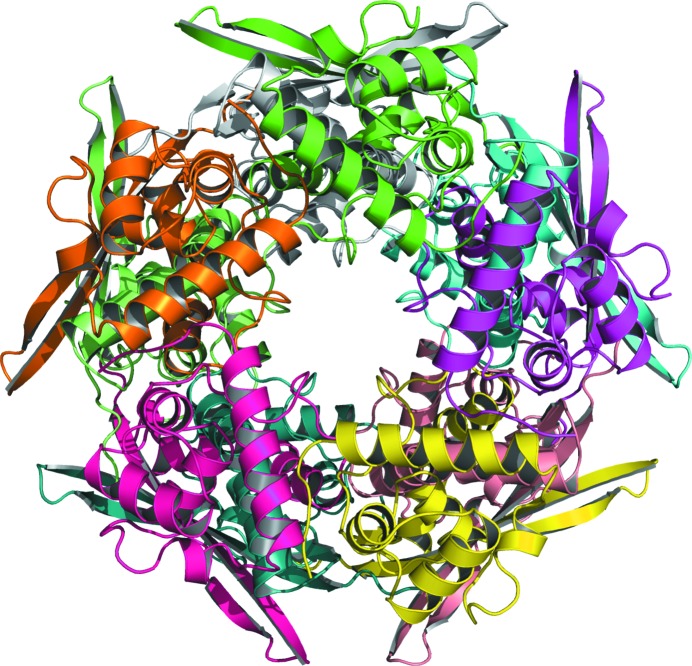
Cartoon representation of the *S. proteamaculans* cyanase decamer, with protomers identified by colour.

The structure of the cyanase was refined with *phenix.refine* (Adams *et al.*, 2010 ▸) to 1.91 Å resolution, yielding *R* and *R*_free_ values of 16.0% and 20.2%, respectively. Manual model inspection and modifications were performed with *Coot*. In the crystal, ten molecules of the protein form a dimeric pentagonal ring (Fig. 5 ▸). The coordinates and structure factors have been deposited in the Protein Data Bank as entry 6b6m.

Following refinement, the cyanase crystallized was found to have the same sequence as PDB entry 4y42 in spite of the fact that the cytokine was produced in an *E. coli* cell line and the receptors were produced in insect cells. This suggests that one of the expression organisms had became contaminated with *S. proteamaculans*, which in turn led to the contaminant crystals.

Both the *SIMBAD* contaminant search and the *ContaMiner* contaminant search allow users to limit the search to common contaminants from a specific host organism. Normally, this is a logical step that saves computing time; however, this case demonstrates the value of making no assumptions where contaminant origin is concerned.

This case also highlighted a limitation in the iteration of the *SIMBAD* lattice-parameter search used. PDB entry 4y42 had been identified as a search model by the lattice-parameter search. However, subsequent MR/refinement failed to provide a solution. Analysis of why PDB entry 4y42 failed to provide a solution at the lattice-parameter stage revealed an oversight in how structures were being input as search models. At the time that this case was run, all models identified by the lattice-parameter search were input into MR with no modifications following download from the PDB. This method had proved sufficient to solve structures which had been crystallized in identical forms to structures already present in the PDB. However, this would break in scenarios where structures were crystallized in symmetry-related space groups.

In this instance, our search model (PDB entry 4y42) was crystallized in space group *P*1 with ten molecules in the asymmetric unit, whereas our crystals had crystallized in space group *C*121 with only five molecules in the asymmetric unit. Using PDB entry 4y42 as a search model without modification led to the failure of MR as the MR search was trying to place too many monomers. *SIMBAD* has subsequently been modified as a result of this case to use a Matthews coefficient to check whether a search model can fit into the asymmetric unit prior to MR. If the full PDB entry is too large to be used as a search model, only the first chain is used. This alteration allowed a solution to be found at the lattice-parameter search instead of the *MoRDa* DB search in subsequent testing.

#### 3.2.3. *E. coli* catalase HPII protein contaminant   

**Figure 6:**
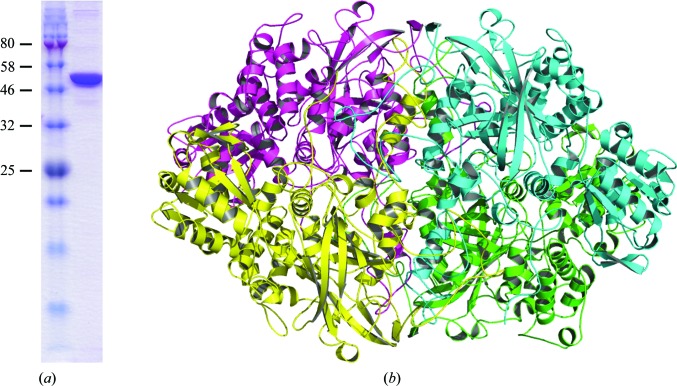
(*a*) SDS–PAGE of the protein sample employed for crystallogenesis. Molecular-mass markers are labelled in kDa. (*b*) Cartoon representation of the *E. coli* catalase HPII tetramer, with protomers identified by colour.

Crystals of the contaminant protein catalase HPII grew from a 10 mg ml^−1^ solution of target protein A (Fig. 6 ▸ *a*). Protein A was produced in *E. coli* TOP10F′ cells, overexpressed as a recombinant fusion with a 6×His tag and purified by successive metal-affinity and size-exclusion chromatography steps. Mass spectrometry (4800 MALDI-TOF/TOF, Abi Sciex) confirmed the anticipated identity of the purified target protein. Crystals were thereafter obtained by vapour diffusion after three months of incubation at 19°C in 600 nl drops composed of a 1:1 mixture of protein and reservoir solutions in a sitting-drop setup with 90 µl reservoir solution [0.085 *M* Na HEPES pH 7.5, 17%(*w*/*v*) PEG 4000, 15%(*v*/*v*) glycerol, 8.5%(*v*/*v*) 2-propanol or 0.1 *M* HEPES pH 7.0, 20%(*w*/*v*) PEG 6000, 1.0 *M* lithium chloride] in the reservoir. Single crystals reached a length of approximately 60 µm and were flash-cooled in liquid nitrogen. X-ray diffraction data were collected on beamline I04 at Diamond Light Source, UK employing radiation of wavelength 0.97946 Å. The diffraction data were processed using *XDS* and scaled with *AIMLESS* (Evans & Murshudov, 2013 ▸) from the *CCP*4 program suite. The crystals grew in space group *P*1, with unit-cell parameters *a* = 69.34, *b* = 90.14, *c* = 114.76 Å, α = 107.10, β = 105.60, γ = 95.98°, and diffracted X-rays to 2.93 Å resolution. Initial phasing attempts failed using molecular replacement (MR) with models displaying 20–30% sequence identity to our target (the best hits available in the PDB) as search probes. *Ab initio*/MR phasing strategies such as *ARCIMBOLDO* also proved to be unsuccessful, likely owing to limited resolution. We decided to optimize the crystallization conditions with the aim of obtaining crystals that diffracted X-rays to higher resolution, as well as to apply experimental phasing methods. Similar crystals did grow in the optimization plates after three-month incubations from 2 µl hanging drops with 1 ml reservoir solution in the reservoir, indicating that crystallogenesis was reproducible, even though new protein batches were used. However, a contaminant search performed at this point with *SIMBAD* readily identified PDB entry 3vu3 (Yonekura *et al.*, 2013 ▸) as a successful MR search model. Four copies of the 84 kDa product of the *E. coli* *katE* gene were found in the *P*1 unit cell, revealing the known homotetrameric assembly of catalase HPII (Fig. 6 ▸ *b*). The structure was refined by iterative cycles of manual model building with *Coot* and refinement with *BUSTER* (Bricogne *et al.*, 2017 ▸), leading to final *R* and *R*_free_ values of 0.183 and 0.236, respectively. Atomic coordinates and structure factors were deposited in the PDB as entry 6by0 and the raw data have been deposited in SBGrid. Mass spectrometry with a Quadrupole-Orbitrap hybrid mass spectrometer (Q-Exactive Plus, Thermo) revealed that the *E. coli* catalase HPII was present in an ∼1:40 ratio relative to our target in the protein samples employed for crystallization. Even though catalase HPII is a known contaminant that is prone to crystallize (Yonekura *et al.*, 2013 ▸), the *P*1 crystal lattice had not previously been reported, escaping a PDB-wide search as demonstrated by the *SIMBAD* lattice-parameter search.

## 4. Discussion   

*SIMBAD* has been designed to be used in a range of different scenarios where conventional sequence-based MR methods have failed. So far, *SIMBAD* has proved to be effective at identifying crystal contaminants, as also have other similar methods such as *MarathonMR* (Hatti *et al.*, 2016 ▸) and *ContaMiner* (Hungler *et al.*, 2016 ▸), suggesting that contamination is one of the main reasons that conventional methods fail. Alongside *MarathonMR* (Hatti *et al.*, 2017 ▸), *SIMBAD* has also proved effective in cases where crystals have been mislabelled. This can happen for various reasons, especially in multi-laboratory collaborations. *SIMBAD* has also successfully determined the structures of unsequenced proteins and a case of swapped crystallization trays (data not shown). More ambitiously, *SIMBAD* also provides a possible means to solve a novel target which is structurally similar to an existing protein in the *MoRDa* DB but whose relationship to that structure is not apparent by sequence comparisons alone.

The different elements of the *SIMBAD* pipeline have very different computational demands. The fastest step in the pipeline is the lattice-parameter search. The experimental lattice parameters are compared with the lattice parameters stored in the Niggli cell database (129 947 at the time of writing) in less than 10 s. Subsequent MR can take as little as 30 s when the top-scoring search model results in a solution and typically less than 15 min for more difficult cases. The next fastest step, the contaminant search, typically runs in about 15 min using four cores (3.2 GHz, Intel i5-6500) when run against the full contaminant database (349 structures and 443 associated *MoRDa* domains at the time of writing). Users can reduce the number of search models to try by specifying the expression organism using the UniProt mnemonic (The UniProt Consortium, 2017 ▸); for example, *E. coli* would be ECOLI whereas *Saccharomyces cerevisiae* (strain ATCC 204508/S288c) would be YEAST. This can improve the speed of the contaminant search, although it could also reduce its effectiveness in cases where the expression organism cell line has become contaminated by a different microorganism. The lattice-parameter and contaminant searches are very quick, and could easily be run routinely after data collection on beamlines to check for the possibility of a contaminant/mislabelled protein. This would allow the identification of a problem and suggest additional data collection from a different crystallization trial when available.

The most time-consuming step in the pipeline is the *MoRDa* DB search. Using a 100-core cluster (2.8 GHz, AMD Opteron 4184) on cases where all 90 000 search models were tried, the *MoRDa* DB search typically took 4–12 h. When fewer than 90 000 search models were tried, the *MoRDa* DB search was significantly quicker. For example, using the 100-core cluster on TOXD (a 59-amino-acid α-dendrotoxin with one molecule in the asymmetric unit that is distributed as an example case by CCP4) took less than an hour as only ∼20 000 search models could potentially fit into the unit cell. Whereas the lattice-parameter search and contaminant search are suitable for use on desktop computers, the *MoRDa* DB search is primarily aimed at clusters. Nonetheless, testing has found that the *MoRDa* DB search can also be run tolerably quickly on a modern multi-core desktop. Using an eight-core/16-thread machine (3.0 GHz, Intel i7-5960X), the *MoRDa* DB search took between 1 and 2 d on a range of test cases where no search models were excluded. The *MoRDa* DB itself requires 2.8 GB of storage.

### 4.1. Future developments   

There are several areas that will be explored in the future to determine whether they improve the effectiveness of *SIMBAD*. A key area will be expanding the database used by the *MoRDa* DB search to also include truncated variants and oligomeric forms of proteins. As the *MoRDa* DB is a reduced database, the top model identified by the *SIMBAD* *MoRDa* DB search will not necessarily be the closest available match in the PDB. Therefore, another area to explore is whether homologues of the best search model which were removed when the redundancy was reduced in the construction of the *MoRDa* DB can provide a better MR solution.

To date, it has been difficult to build an accurate picture of common contaminants, as these structures often go either unsolved or unpublished.

As *SIMBAD* becomes used more regularly we foresee the possibility of gathering significantly more data on common contaminants and therefore improving our contaminant database. We are also developing *SIMBAD* to use ContaBase (provided by *ContaMiner*) as a source to update our contaminant database. Therefore, in the event that a user identifies a novel contaminant, we suggest submitting the contaminant to ContaBase, where it will benefit both future *SIMBAD* and *ContaMiner* searches.

Another avenue to explore is whether alternative scoring systems increase the effectiveness of *SIMBAD*, as might alternative MR programs for the rotation search in place of the current Patterson-based *AMoRe* search. In particular, we plan to explore the maximum-likelihood-based rotation search in *Phaser* using its convenient capacity to process a batch of search models in a single job. Of key interest will be how other MR programs affect the sensitivity of the pipeline and its speed.

Currently, the lattice-parameter search and contaminant search are available in *CCP*4*i* and *CCP*4*i*2 on *nix-based architectures, with plans to bring *SIMBAD* to *CCP*4 online services.

## 5. Conclusions   

Crystal contamination is a possibility that every crystallo­grapher should bear in mind when performing an experiment. *SIMBAD* provides a rapid and reliable means to check for the presence of a contaminant. *SIMBAD* is also useful in cases of the misidentification of a crystal and can also be useful in scenarios where no obvious homologue is available as a search model or the most suitable search model is not among those most highly ranked by sequence comparisons. The lattice-parameter and contaminant searches in *SIMBAD* are very quick, and we therefore suggest running them routinely after data collection on beamlines to identify possible cases of contaminant crystallization or protein mislabelling.

## Supplementary Material

PDB reference: *Escherichia coli* DPS, 6b0d

PDB reference: *E. coli* catalase HPII, 6by0

PDB reference: *Serratia proteamaculans* cyanase, 6b6m

Supplementary Figure S1.. DOI: 10.1107/S2059798318005752/rr5159sup1.pdf

Supplementary Table S1. The 125 randomly selected data sets used to calculate the probability score for the lattice-parameter search.. DOI: 10.1107/S2059798318005752/rr5159sup2.xlsx

Supplementary Table S2. Classification reports for the training set and the test set of the probability model. Generated using scikit-learn (http://www.scikit-learn.org).. DOI: 10.1107/S2059798318005752/rr5159sup3.xlsx

Supplementary Table S3. Results from the contaminant testing.. DOI: 10.1107/S2059798318005752/rr5159sup4.xlsx

Supplementary Table S4. Results from the lattice and MoRDa DB testing.. DOI: 10.1107/S2059798318005752/rr5159sup5.xlsx
