# Supplementary material for: SIMBAD: a sequence-independent molecular-replacement pipeline
Source: Acta Crystallogr D Struct Biol. 2018 Jun 8;74(Pt 7):595–605. doi: 10.1107/S2059798318005752 (PMC6038384; doi:10.1107/S2059798318005752)
Supplement: Supplementary file 1 [file d-74-00595-sup1.pdf]

STRUCTURAL
BIOLOGY

Volume 74 (2018)

Supporting information for article:

SIMBAD: a sequence-independent molecular-replacement pipeline

Adam J. Simpkin, Felix Simkovic, Jens M. H. Thomas, Martin Savko, Andrey Lebedev, Ville Uski, Charles Ballard, Marcin Wojdyr, Rui Wu, Ruslan Sanishvili, Yibin Xu, María-Natalia Lisa, Alejandro Buschiazzo, William Shepard, Daniel J. Rigden and Ronan Keegan

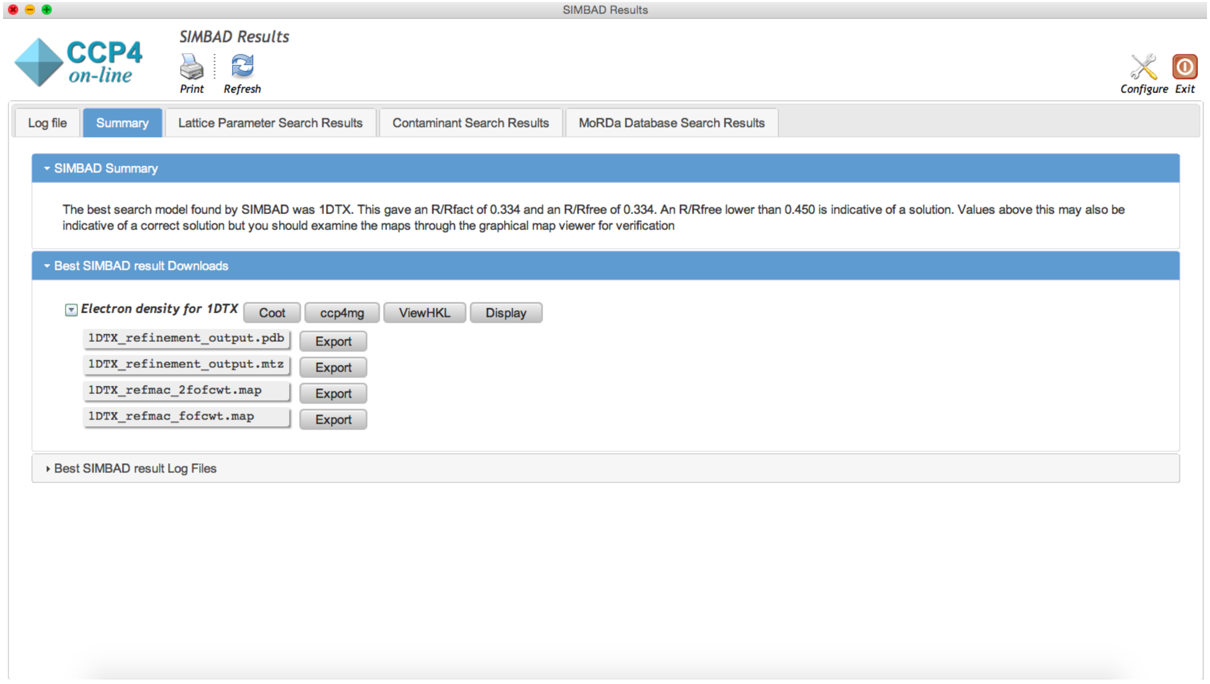

Figure S1 A *SIMBAD* report page showing a summary tab from a *SIMBAD* run.
